# Class III *β*-tubulin expression and *in vitro* resistance to microtubule targeting agents

**DOI:** 10.1038/sj.bjc.6605489

**Published:** 2009-12-22

**Authors:** C Stengel, S P Newman, M P Leese, B V L Potter, M J Reed, A Purohit

**Affiliations:** 1 Oncology Drug Discovery and Women's Health Group, Faculty of Medicine, Imperial College London, St Mary's Hospital, London W2 1NY, UK; 2 Medicinal Chemistry, Department of Pharmacy and Pharmacology and Sterix Ltd, University of Bath, Bath, UK

**Keywords:** class III *β*-tubulin, microtubule disruptor, paclitaxel, STX140, MCF-7 cells, MDA-MB-231 cells

## Abstract

**Background:**

Class III *β*-tubulin overexpression is a marker of resistance to microtubule disruptors *in vitro*, *in vivo* and in the clinic for many cancers, including breast cancer. The aims of this study were to develop a new model of class III *β*-tubulin expression, avoiding the toxicity associated with chronic overexpression of class III *β*-tubulin, and study the efficacy of a panel of clinical and pre-clinical drugs in this model.

**Methods:**

MCF-7 (ER+ve) and MDA-MB-231 (ER−ve) were either transfected with pALTER-TUBB3 or siRNA-tubb3 and 24 h later exposed to test compounds for a further 96 h for proliferation studies. RT–PCR and immunoblotting were used to monitor the changes in class III *β*-tubulin mRNA and protein expression.

**Results:**

The model allowed for subtle changes in class III *β*-tubulin expression to be achieved, which had no direct effect on the viability of the cells. Class III *β*-tubulin overexpression conferred resistance to paclitaxel and vinorelbine, whereas downregulation of class III *β*-tubulin rendered cells more sensitive to these two drugs. The efficacy of the colchicine-site binding agents, 2-MeOE2, colchicine, STX140, ENMD1198 and STX243 was unaffected by the changes in class III *β*-tubulin expression.

**Conclusion:**

These data indicate that the effect of class III *β*-tubulin overexpression may depend on where the drug’s binding site is located on the tubulin. Therefore, this study highlights for the first time the potential key role of targeting the colchicine-binding site, to develop new treatment modalities for taxane-refractory breast cancer.

The high percentage of non-responders and failures following initial responses to taxanes (taxotere and paclitaxel) highlights the critical role played by drug resistance mechanisms in breast cancer progression. Although many mechanisms associated with resistance have been proposed for taxane-refractory cells, only two have been found in the clinic to date: the overexpression of ABC proteins such as the P-glycoprotein (MDR1) and alterations in tubulin-isoform expression (Correnti *et al*, 1995; Linn *et al*, 1995; Kavallaris *et al*, 1997; Mozzetti *et al*, 2005; Paradiso *et al.*, 2005). Mutations within tubulin at the paclitaxel-binding site have been identified (Giannakakou *et al*, 1997), which were thought to be associated with taxane resistance. However, subsequent studies showed that these early findings resulted from the amplification of tubulin pseudogenes (Noguchi, 2006). Further studies have failed to confirm the presence of tubulin point mutations in patients with lung or ovarian cancers resistant to therapy (Sale *et al*, 2002; Tsurutani *et al*, 2002; Lamendola *et al*, 2003). Microtubule-targeting agents bind to the *β*-tubulin subunit of the *α*/*β*-tubulin heterodimers that assemble to form microtubules. In humans, at least seven distinct *β*-tubulin isotypes have been reported. Altered patterns of expression are seen in cancer. In ovarian cancer patients class III *β*-tubulin mRNA is significantly upregulated in the taxane-resistant tumours compared with biopsy samples from untreated tumours (Kavallaris *et al*, 1997). In a separate study, class III *β*-tubulin mRNA and protein were shown to be significantly upregulated in a sub-set of paclitaxel resistant ovarian cancer patients (Mozzetti *et al*, 2005). In breast cancer high expression of class III *β*-tubulin is a predictive biomarker of clinical paclitaxel resistance (Tommasi *et al*, 2007). In gastric cancer patients, whose tumours were positive for class III *β*-tubulin expression were significantly less likely to respond to docetaxel-based chemotherapy, 16.7 *vs* 64.3% response rate, respectively. Therefore, class III *β*-tubulin can be regarded as a predictive marker for the clinical response to docetaxel-based chemotherapy in gastric cancer (Urano *et al*, 2006). The ongoing development of new agents, not sensitive to class III *β*-tubulin overexpression, may provide new treatment options for treatment-refractory cancer.

In this study, the effects of seven microtubule targeting agents on the proliferation of MCF-7 and MDA-MB-231 breast carcinoma cells over- or underexpressing class III *β*-tubulin were investigated. Five of the agents, 2-methoxyoestradiol-3,17-*O*, *O-bis*-sulfamate (STX140), 2-ethyloestradiol-3,17-*O,O*-*bis*-sulfamate (STX243), 2-methoxyoestradiol (Panzem), colchicine and ENMD1198 are known to target the colchicine-binding site on tubulin, the other two target the taxane-binding site (paclitaxel) and the vinca-binding site (vinorelbine). These seven agents represent the currently used drugs in the clinic (paclitaxel and vinorelbine), those drugs that are currently in/have recently completed phase I trials (2-MeOE2 and ENMD1198) and a new generation of orally bioavailable compounds which are in advanced pre-clinical development (STX140 and STX243).

## Materials and methods

### Drug synthesis

**Figure 1:**
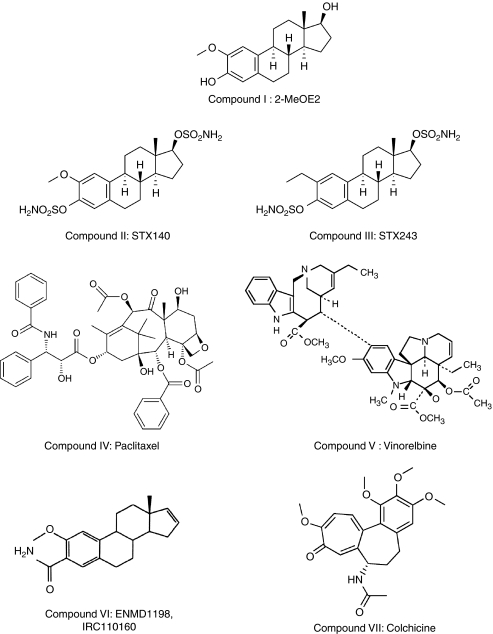
Structures. I: 2-MeOE2, II: STX140, III: STX243, IV: paclitaxel, V: vinorelbine, VI: ENMD1198 and VII: colchicine.

2-Methoxyoestradiol (2-MeOE2, Figure 1 compound I) was synthesised as described previously (Leese *et al*, 2005a). 2-Methoxyoestradiol-3,17-*O*,*O*-*bis*-sulfamate (STX140, Figure 1 compound II) and 2-ethyloestradiol-3,17-*O,O*-*bis*-sulfamate (STX243, Figure 1 compound III) were synthesised by reaction of the appropriate 2-substituted oestradiol in dimethyl acetamide solution with sulphamoyl chloride (Leese *et al*, 2005b, 2006). 2-Methoxyoestra-1,3,5(10),16-tetraene-3-carboxamide (ENMD1198, IRC110160, Figure 1 compound VI) was synthesised as described in US2005/203075 (Foster *et al*, 2008). Paclitaxel (Figure 1 compound IV, Sigma, Poole, UK), vinorelbine (Figure 1 compound V, Sigma) and colchicine (Figure 1 compound VII, Sigma) were purchased from commercial sources.

### Cell culture

MCF-7 (ER+ve) and MDA-MB-231 (ER−ve) human breast cancer cells were obtained from the American Type Culture Collection (LGC Promochem, Teddington, UK). Cells were cultured in RPMI 1640 medium supplemented with 10% (v/v) foetal calf serum, 1% L-glutamine (200 mM), 1% non-essential amino acids (100 × ) and 1% bicarbonate (7.5%) from Sigma and maintained in a humidified incubator under 5% CO_2_ atmosphere at 37°C.

### Class III *β*-tubulin cloning

The protein-expression vector (pALTER-TUBB3) was prepared by inserting the full-length human class III *β*-tubulin cDNA fragment from pOTB7-TUBB3 (LGC Promochem) into pALTER Max (Promega, Southampton, UK). The construct was checked by digestion and sequencing.

### Transfection

MCF-7 and MDA-MB-231 cells were transfected with the wild-type class III *β*-tubulin (pALTER-TUBB3) or with the class III *β*-tubulin siGenome Smartpool (siRNA-tubb3, ref. M-020099-03, Dharmacon, Cramlington, UK) using the Amaxa nucleofection technology (Amaxa GmbH, Koeln, Germany). The nucleofaction protocol for plasmid transfection was optimised using a GFP-construct with efficacies of 47 and 70% being achieved after 24 h transfection in MCF-7 and MDA-MB-231 cells, respectively. siRNA nucleofection was carried out using a pre-optimised protocol (Mhaidat *et al*, 2008). Briefly, the cells were trypsinised and washed with PBS. The required number of cells (2 × 10^6^ cells per nucleofection) were harvested, resuspended in nucleofactor solution (100 *μ*l per nucleofection) and mixed with 2 *μ*g plasmid or 4 *μ*l of siRNA (20 *μ*M). Cells were then electroporated in the Amaxa nucleofector and transferred to fresh pre-warmed medium. The cells were incubated for 1 h at 37°C before being seeded in 96-well plates for proliferation assay or in T-25 flasks for protein or RNA extraction. Transfection with the empty pALTER-Max (mock transfection) and a non-targeting siRNA (siGENOME Non-targeting siRNA, ref. D-001210-01, Dharmacon) had no significant effect on the proliferation of either cell line (data not shown).

### Proliferation assay

The cells were seeded into 96-well culture plates at 4000 cells per well, incubated at 37°C for 24 h and exposed to compounds for 96 h at 37°C. Cell proliferation was measured by adding 10 *μ*l AlamarBlue reagent (BioSource International, Camarillo, CA, USA). The viable cells are counted with a spectrofluorometric microtiter-well plate reader Fluostar optima (BMG Labtech GmbH, Offenburg, Germany) at 544 nm (excitation) and 590 nm (emission). IC_50_ values were obtained from a graph of percent proliferation *vs* inhibitor concentration and calculated using Prism (Graphpad Software, La Jolla, CA, USA).

### Immunoblotting

Proteins were extracted from the transfected cells by homogenisation in RIPA buffer (Sigma) in the presence of 10% protease inhibitors and phosphatase inhibitors (ref. P2714, P5726 and P2850; Sigma). The extracts were centrifuged (15 min at 16 000 **g** at 4°C) and total protein concentrations were determined by Bradford assay (Sigma). Protein samples (50 *μ*g) were denatured at 70°C for 10 min in loading buffer, separated by electrophoresis using 4–12% Bis-Tris Nupage gels (Invitrogen, Paisley, UK) and blotted onto a PVDF membrane (GE Healthcare, Bucks, UK). Successful transfer and even protein loading was confirmed by staining with Ponceau S Red (ref. 09189; Sigma). Only membranes with even protein loading were further processed (data not shown). The membrane was blocked with blocking buffer (4% milk in PBS-Tween 1%) for 2 h and incubated overnight at 4°C with the anti-human class III *β*-tubulin antibody (ref. MAB1637; Millipore, Watford, UK) at 1 : 1000 or anti-human class I–V *β*-tubulin antibody (ref. T4026; Sigma) at 1 : 200. The anti-human *α*-tubulin was used as loading control at 1 : 1000 (ref. Ab6161; Abcam, Cambridge, UK). An alkaline phosphatase-conjugated anti-mouse (ref. 7056; Cell Signaling, Beverly, MA, USA) was used as secondary antibody and the immunoblot was developed with a chemifluorescent substrate (ECF substrate, ref. RPN5785; GE Healthcare) and visualised using a Storm imaging system (GE Healthcare). The data shown are representative of one of three such experiments.

### Real time RT–PCR

Cells were lysed with 350 *μ*l of RNeasy lysis buffer (Qiagen, Ulm, Germany) plus 1% *β*-mercaptoethanol (Promega) and homogenised with the QIAshredder columns (Qiagen). The RNA was isolated from the homogenate using the RNeasy kit (Qiagen).

A 5 *μ*g aliquot of each RNA sample was reverse transcribed using the First-Strand cDNA Synthesis kit (GE Healthcare). Reverse-transcription–PCRs were carried out in a Rotor Gene 2000 Real-time Cycler (Corbett Research, Cambridge, UK) with 2 *μ*l cDNA in a final volume of 25 *μ*l using Taqman Universal PCR Master Mix (Applied Biosystems, Foster City, CA, USA). Primers and hydrolysis probes for class III *β*-tubulin (ref. Hs00964965_m1) and for the internal housekeeping gene, *RPLO*, (ref. 4310879E) were synthesised by Applied Biosystems. The relative Ct values were calculated using the Rotor Gene 6 software (Corbett Research, Cambridge, UK) and the comparative Cts were given as: 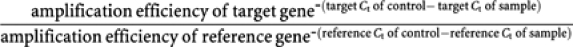


### Immunohistochemistry

MCF-7 and MDA-MB-231 cells grown on poly-L-lysine-coated coverslips were exposed to compounds for 24 h. The cells were fixed in methanol at −20°C for 10 min, and then in ice-cold acetone for 30 s. Coverslips were rinsed and then rehydrated with PBS. To visualise the microtubules, cells were incubated for 1 h with an *α*-tubulin-specific FITC-conjugated antibody (ref. F2168, clone DM1A; Sigma) in PBS containing 1% BSA. Cells were washed twice with PBS and counterstained with Hoechst 33342 at 1 mg ml^−1^ (Sigma) to stain the nuclei. The coverslips were mounted on slides using DePex mounting medium, observed using a Zeiss inverted microscope ( × 200) and analysed with the Axiovision imaging system.

### Statistics

*In vitro* experiments were carried out in triplicate and data presented are representative of three or more independent experiments. All errors shown are the mean±s.d. The Student's *t*-test was used to assess significance.

## Results

### Class III *β*-tubulin mRNA expression and protein expression

**Figure 2:**
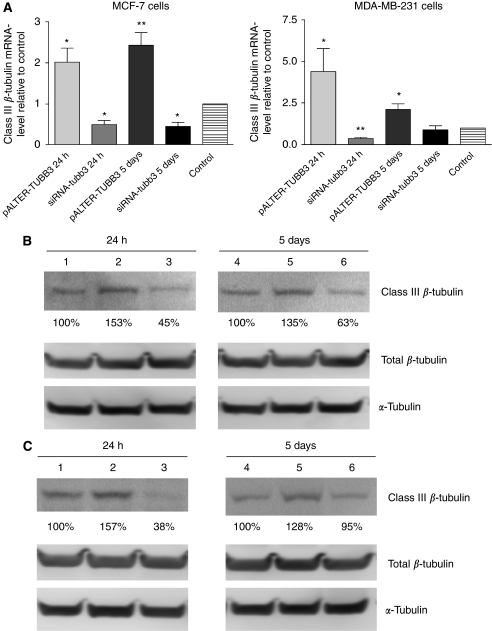
Class III *β*-tubulin mRNA and protein expression. (**A**) MCF-7 and MDA-MB-231 cells were transfected with the mock vector or the non-targeting siRNA (control), pALTER-TUBB3 or siRNA-tubb3 and the expression of class III *β*-tubulin mRNA was quantified by RT–PCR. Three or four independent experiments (*n*=3–4) were carried out in duplicate and the results presented are the average of the Ct values relative to the control Ct values, bars: mean±s.d., ^*^P<0.05 and ^**^P<0.01. The expression of class III *β*-tubulin protein, total *β*-tubulin and total *α*-tubulin was analysed in (**B**) MCF-7 and (**C**) in MDA-MB-231 24 h and 5 days after transfection by SDS–PAGE and immunoblotting. (1) Control 24 h, (2) pALTER-TUBB3 24 h, (3) siRNA-tubb3 24 h, (4) control 96 h, (5) pALTER-TUBB3 96 h and (6) siRNA-tubb3 96 h.

MCF-7 and MDA-MB-231 cells were transfected with mock vector, non-targeting siRNA (MCF-7-control and MDA-MB-231-control), pALTER-TUBB3 (MCF-7-TUBB3 and MDA-MB-231-TUBB3) or siRNA-tubb3 (MCF-7-situbb3 and MDA-MB-231-situbb3). Gene expression levels of class III *β*-tubulin were examined by RT–PCR. The mRNA level of class III *β*-tubulin was 2±0.76-fold increased in MCF-7-TUBB3 cells 24 h after transfection (*P*<0.05) and 2.4±0.68-fold increased 5 days after transfection (*P*<0.01), compared with MCF-7-mock-transfected cells (Figure 2A). The mRNA level of class III *β*-tubulin was 4.4±2.6-fold increased in MDA-MB-231-TUBB3 cells 24 h after transfection (*P*<0.05) and was 2.1±0.74-fold higher than the control level 5 days after transfection (*P*<0.05, Figure 2B) relative to MDA-MB-231 mock-transfected cells. Transfection with siRNA-tubb3 blocked the class III *β*-tubulin mRNA expression in both cell lines (Figure 2A and B). Class III *β*-tubulin mRNA levels were reduced by 50±16% in MCF-7-situbb3 cells 24 h after transfection (*P*<0.05) and by 46±16% 5 days after transfection (*P*<0.05). Class III *β*-tubulin mRNA levels were also reduced by 36±15% in MDA-MB-231-situbb3 cells 24 h after transfection (*P*<0.01), but were not significantly different from control cells 5 days after transfection (90±47%).

To ensure changes in mRNA level correlated with protein expression, MCF-7 and MDA-MB-231 cells were transfected with either pALTER-TUBB3 or siRNA-tubb3 and the proteins were extracted 24 h and 5 days after transfection. Equal protein amounts were analysed by SDS–PAGE and immunoblotting with the human anti-class III *β*-tubulin or the human anti-class I–V *β*-tubulin. Staining with Ponceau S and immunoblotting with an anti-*α*-tubulin was used to ensure even protein loading and transfer. Class III *β*-tubulin protein expression was higher in both cell lines transfected with the pALTER-TUBB3 vector (Figure 2B and C, lane 2) and was lower in cells transfected with the class III *β*-tubulin siRNA (Figure 2B and C, lane 3) compared with the control cells (Figure 2B and C, lane 1). These changes in protein expression were still observed 5 days after transfections (Figure 2B and C, lanes 4, 5 and 6), except for MDA-MB-231 transfected with siRNA-tubb3. The transfection with either pALTER-TUBB3 or with the siRNA did not affect the amount of total *β*-tubulin expressed in cells (Figure 2B and C). No change was observed for cells transfected with the non-targeting siRNA (data not shown).

### Proliferation assays

**Figure 3:**
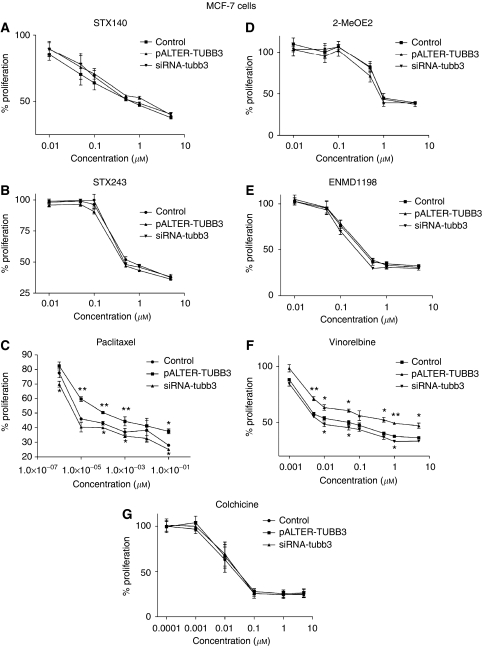
STX140, STX243, 2-MeOE2, paclitaxel, ENMD1198, vinorelbine and colchicine chemosensitivity in MCF-7 cells. MCF-7 cells were transfected with the mock vector or the non-targeting siRNA (control), (**A**) pALTER-TUBB3 or siRNA-tubb3 and exposed to STX140, (**B**) STX243, (**C**) paclitaxel, (**D**) 2-MeOE2, (**E**) ENMD1198, (**F**) vinorelbine or (**G**) colchicine for 4 days. The percent proliferation was determined by AlamarBlue assay and results presented are the average of three or four independent experiments (*n*=3–4) done in triplicate, ^*^P<0.05 and ^**^P<0.01.

**Figure 4:**
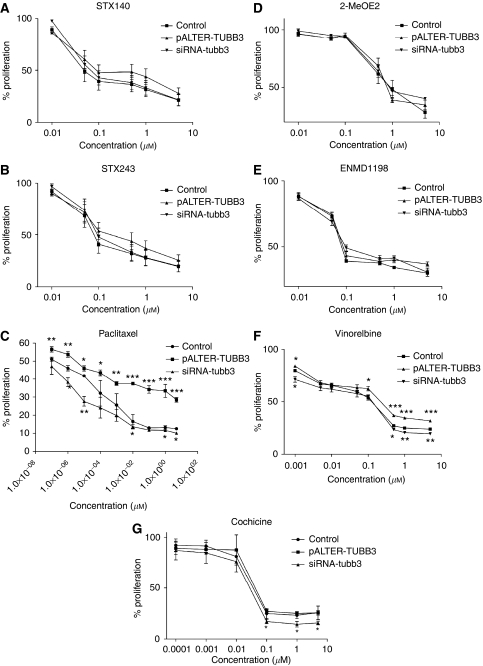
STX140, STX243, 2-MeOE2, paclitaxel, ENMD1198, vinorelbine and colchicine chemosensitivity in MDA-MB-231 cells. MDA-MB-231 cells were transfected with the mock vector or the non-targeting siRNA (control), (**A**) pALTER-TUBB3 or siRNA-tubb3 and treated with STX140, (**B**) STX243, (**C**) paclitaxel, (**D**) 2-MeOE2, (**E**) ENMD1198, (**F**) vinorelbine or (**G**) colchicine for 4 days. The percent proliferation was determined by AlamarBlue assay and results presented are the average of three or four independent experiments (*n*=3–4) done in triplicate, ^*^P<0.05, ^**^P<0.01 and ^***^P<0.001.

MCF-7 and MDA-MB-231 transfected cells were seeded at 4000 cells per well, incubated for 4 days and the percentage of proliferation was monitored by using the Alamar blue reagent. Class III *β*-tubulin overexpression or silencing did not significantly affect the viability of the cells (the proliferation of MCF-7-TUBB3, MCF-7-situbb3, MDA-MB-231-TUBB3 and MDA-MB-231-situbb3 cells was 105, 101, 97 and 98% respectively, the proliferation rate of control cells, ns). The cytotoxicity efficacy of STX140, STX243, 2-MeOE2, ENMD1198 and colchicine was not significantly affected by overexpression or silencing of class III *β*-tubulin (Figures 3 and 4, graphs A, B, D, E and G), and the IC_50_ values of these compounds were not significantly different from IC_50_ values calculated in control cells (Table 1a and b). Although MDA-MB-231 cells underexpressing the class III *β*-tubulin were more sensitive to colchicine than the mock-transfected cells when exposed to high concentrations (0.1–10 *μ*M), the IC_50_ values in both type of cells were not significantly different (IC_50_=37 nM in MDA-MB-231-mock and IC_50_=29 nM in MDA-MB-231-situbb3, Table 1b).

Class III *β*-tubulin expression altered the sensitivity to paclitaxel in both cell lines (Figures 3C and 4C). MCF-7 and MDA-MB-231 cells overexpressing class III *β*-tubulin were the less sensitive to paclitaxel, with IC_50_ values of 0.1 nM and 4 pM, respectively (Table 1a and b). These IC_50_ values were significantly higher (*P*<0.01) than those obtained in untreated MCF-7 and MDA-MB-231 cells (7.2 and 2 pM, respectively). In contrast, MCF-7 and MDA-MB-231 cells underexpressing class III *β*-tubulin were found to be the most sensitive to paclitaxel. Paclitaxel's IC_50_ value was significantly reduced in MCF-7-situbb3 cells (IC_50_=4.8 pM, *P*<0.05) compared to the IC_50_ value in control cells. Despite reducing the paclitaxel concentration to 0.1 pM, no IC_50_ values could be obtained for the MDA-MB-231 cells underexpressing class III *β*-tubulin as the inhibition of growth was greater than 50% (47%, Figure 4C). At higher concentrations (1 and 10 pM), the class III *β*-tubulin silencing rendered MDA-MB-231 cells significantly more sensitive to paclitaxel than the control cells (*P*<0.05 and *P*<0.01, respectively, Figure 4C).

The modification of the *β*-tubulin expression profile also altered the efficacy of vinorelbine (Figures 3F and 4F). When MCF-7 and MDA-MB-231 cells were transfected with pALTER-TUBB3, the IC_50_ values for vinorelbine were higher than the IC_50_ values in the control cells (the IC_50_ values were 1 *μ*M in MCF-7-TUBB3 and 0.25 *μ*M in MDA-MB-231-TUBB3 cells, whereas they were equal to 0.06 *μ*M in MCF-7-control and 0.12 *μ*M in MDA-MB-231-control cells, Table 1a and b). The MCF-7 cells transfected with class III *β*-tubulin silencing RNA were the most sensitive to vinorelbine and their IC_50_ value was equal to 0.01 *μ*M. Underexpression of class III *β*-tubulin did not affect the sensitivity of MDA-MB-231 cells to vinorelbine and the IC_50_ value in these cells remained the same as in control cells (IC_50_=0.12 *μ*M in MDA-MB-231-situbb3).

### Microtubule structure

**Figure 5:**
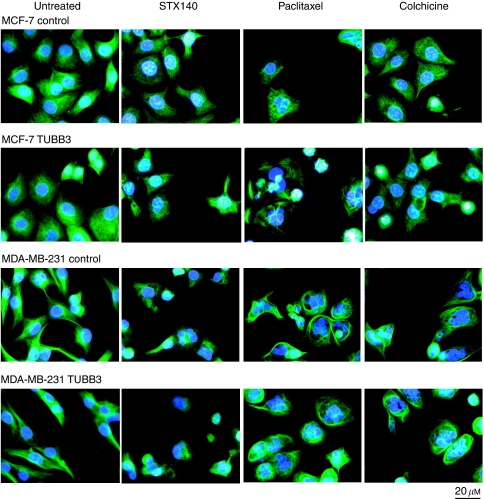
Fluorescent images of cells overexpressing class III *β*-tubulin after drug exposure. MCF-7-control, MCF-7-TUBB3, MDA-MB-231-control and MDA-MB-231-TUBB3 cells were treated for 48 h with STX140, paclitaxel or colchicine at concentrations close to IC_50_ values obtained in control cells and stained with FITC-anti-tubulin (tubulin) and Hoechst 33342 (nucleus). Images were taken using a Zeiss inverted microscope at × 200 magnification.

To investigate whether class III *β*-tubulin resistance is reflected at the cytoskeleton structure level, cells overexpressing class III *β*-tubulin were exposed to STX140, paclitaxel and colchicine for 48 h at concentrations close to IC_50_ values obtained in control cells. Nuclear and cytoskeletal morphology was then examined by immuno-fluorescence microscopy. The untreated cells showed typical nuclear and cytoskeleton structures, with long and dense microtubules extending throughout the cytoplasm. No significant difference was observed in the morphology of both untreated cell lines overexpressing class III *β*-tubulin compared with control cells (Figure 5). STX140 and colchicine caused microtubule disruption with bundles of microtubules being observed and overexpression of class III *β*-tubulin did not affect the morphological changes in either cell line in response to these compounds. In the non-transfected MCF-7 control cells paclitaxel caused the virtual destruction of the normal microtubule structure with only very small and dense bundles of microtubules being visible. Furthermore, many abnormal nuclei were observed in comparison to untreated cells and those exposed to either STX140 or colchicine. Morphological changes were seen in the MCF-7 class III *β*-tubulin overexpressing cells in response to paclitaxel, with shortened but unusually highly defined microtubules and many abnormal nuclei present. The unusual microtubule structures seen in the MCF-7 class III *β*-tubulin overexpressing cells in response to paclitaxel were not observed in their MDA-231 counterparts which far more closely resemble the non-transfected MDA-231 control cells after paclitaxel exposure. Although tubulin III transfection renders both cells lines significantly more resistant to paclitaxel, effects on microtubule structure were seen in this study as the concentration of paclitaxel used was still cytotoxic. Further studies are planned to understand the unusual structures observed in the MCF-7 class III *β*-tubulin overexpressing cells.

## Discussion

Tubulin-binding agents constitute an important class of compound in chemotherapy, but their clinical success has been compromised by the emergence of drug resistance, which is derived from several mechanisms that can be *de novo* or acquired. Tubulin-binding agents target the microtubule network and two different resistance mechanisms have been associated to the taxane-resistant phenotype in cancer patients: the overexpression of ATP-binding cassette family of proteins such as P-glycoprotein and alterations in tubulin-isoform expression. Previous studies showed that some compounds such as STX140, are able to inhibit the proliferation of resistant MCF-7dox cells (P-gly +ve) by arresting them in the G2/M phase (Suzuki *et al*, 2003) and to block the growth of MCF-7dox tumours (Newman *et al*, 2008). This study is the first to report the effects of changes in the tubulin-isoform expression profile on the efficacy of new tubulin-binding agents, including Panzem (2-MeOE2), ENMD1198 (currently in phase I clinical trials) and STX140 in MCF-7 and MDA-MB-231 cells.

Identifying the role of class III *β*-tubulin in drug resistance is difficult because of the complexity of tubulin auto-regulation. Expression of *β*-tubulin isotypes is tightly regulated through co-translational degradation of *β*-tubulin mRNAs in response to an increase in the level of soluble tubulin (Yen *et al*, 1988; Theodorakis and Cleveland, 1992). This may explain why overexpression of class III *β*-tubulin failed to confer resistance to paclitaxel, estramustine and vinblastine, among other drugs tested in prostate cells (Ranganathan *et al*, 2001). In addition, too high expression of class III *β*-tubulin in cells can be toxic, further complicating this methodology (Hari *et al*, 2003). To negate this problem Hari *et al* (2003) inducibly overexpressed class III *β*-tubulin in CHO cells, this decreased the paclitaxel suppression of microtubule dynamics and caused a limited resistance to paclitaxel (Kamath *et al*, 2005). Recently, cervical carcinoma HeLa cells were stably transfected with class III *β*-tubulin (Joe *et al*, 2008) and were used to test taccalonolides, which were shown not to be susceptible to class III *β*-tubulin resistance (Risinger *et al*, 2008). In this study, class III *β*-tubulin protein levels was only 1.5-fold increased in cells transfected with pALTER-TUBB3. This may have protected the cells against the toxicity of high expression and enabled them to grow with a similar proliferation rate than control cells. However, the overexpression of class III *β*-tubulin was significant and this degree of overexpression was sufficient to confer a resistance to paclitaxel. These data are supported by the relatively small increase (five-fold) seen in patients with docetaxel-refractory breast cancer (Hasegawa *et al*, 2003).

This study demonstrates that a variation in the *β*-tubulin expression pattern in two breast cancer cell lines does not affect the efficacies of STX140, STX243, 2-MeOE2, ENMD1198 and colchicine, whereas the efficacy of vinorelbine and paclitaxel are altered by both class III *β*-tubulin overexpression and silencing. These results correlate with previous studies using siRNA approaches which showed that class III *β*-tubulin induces resistance to paclitaxel, vincristine and DNA-damaging agents in non-small cell lung carcinoma (Kavallaris *et al*, 1999; Gan *et al*, 2007).

The key difference between class I and class III *β*-tubulin is an amino-acid substitution leading to a different three dimensional conformation. Class III *β*-tubulin, which presents only 92% similarity with the other *β*-tubulin isoforms, bears an Arg^277^ instead of the Ser^277^ present in class I *β*-tubulin. Ser^277^ and Arg^278^ are essential for the stable binding of paclitaxel to its binding site in class I *β*-tubulin (Lowe *et al*, 2001). In contrast, the structure of the loop of the class III *β*-tubulin is altered by the substitution of Ser^277^ with Arg and thus, prevents a stable paclitaxel binding (Ferlini *et al*, 2005). It has been shown that Class III *β*-tubulin also generates more dynamic microtubules and counteracts pro-assembling activity of taxanes at the plus ends of microtubules (Kamath *et al*, 2005), thereby making microtubules more resistant to the stabilisation of microtubule dynamics operated by taxanes (Hari *et al*, 2003; Kamath *et al*, 2005; Gan *et al*, 2007). In contrast, STX140, STX243, 2-MeOE2, ENMD1198 and colchicine are structurally different to paclitaxel; they bind to the colchicine site and they have a conformation, which may allow the formation of a stable complex with class III *β*-tubulin. Moreover, these agents depolymerise microtubules, whereas paclitaxel stabilises them. This may be a great advantage when class III *β*-tubulin is overexpressed and the microtubule dynamic is amplified. However, vinorelbine, which is also a microtubule-destabilising agent but binds to the *Vinca* alkaloid binding site, has its efficacy reduced by the overexpression of class III *β*-tubulin *in vitro* in breast carcinoma cells (as shown in this study) and in small lung cancer cells (Gan *et al*, 2007) and *in vivo* (Seve *et al*, 2005). Therefore, class III *β*-tubulin resistance might be binding-site related and agents binding to the colchicine-binding site might avoid this resistance. This theory is supported by a recent study, wherein the authors showed that HeLa cells overexpressing class III *β*-tubulin are less sensitive to paclitaxel, docetaxel, epothilone B and vinblastine. Furthermore, the efficacy of the taccalonolides, which do not interact with microtubules despite having paclitaxel-like effects in cells, is unaffected by class III *β*-tubulin expression (Risinger *et al*, 2008).

In summary, this study shows that microtubule disrupting agents binding to the colchicine-binding site circumvent class III *β*-tubulin resistance, whereas agents binding to the taxol- or the *Vinca*-binding sites have their efficacies altered by changes in class III *β*-tubulin expression. Class III *β*-tubulin is associated with drug resistance and poor prognosis in patients (Hasegawa *et al*, 2003; Mozzetti *et al*, 2005; Seve *et al*, 2005; Urano *et al*, 2006; Tommasi *et al*, 2007) and it is a predictive factor of event-free and overall survival (Seve and Dumontet, 2008). Moreover, class III *β*-tubulin is involved in the sensitivity of both taxanes and *Vinca* alkaloids, but it also has a role in chemosensitivity of DNA-damaging agents (Gan *et al*, 2007). This study therefore highlights the importance of developing new agents binding to the colchicine-binding site, such as STX140 and STX243 as an alternative to taxanes for refractory cancer treatment.

## Figures and Tables

**Table 1:** Average IC_50_ values of STX140, STX243, STX66, paclitaxel, ENMD1198, vinorelbine and colchicine in (a) MCF-7 and (b) MDA-MB-231 cells

**MCF-7 cells Avg IC_50_ values (*μ*M)**	**Mock**	**pALTER-TUBB3**	**siRNA-tubb3**
(a)			
STX140	0.98	1.06	0.92
STX243	0.69	0.81	0.73
2-MeOE2	0.99	0.97	0.98
Paclitaxel	7.2 × 10^−6^	1 × 10^−4^^**^	4.8 × 10^−6^^*^
Vinorelbine	0.06	1^**^	0.01^*^
ENMD1198	0.57	0.61	0.45
Colchicine	0.024	0.029	0.029
			
**MDA-MB-231 cells Avg IC_50_ values (*μ*M)**	**Mock**	**pALTER-TUBB3**	**siRNA-tubb3**
(b)			
STX140	0.05	0.08	0.07
STX243	0.08	0.10	0.09
2-MeOE2	0.99	0.98	0.99
Paclitaxel	2 × 10^−6^	4 × 10^−6^^***^	<1 × 10^−7^^**^
Vinorelbine	0.12	0.25^**^	0.12
ENMD1198	0.08	0.09	0.09
Colchicine	0.037	0.042	0.029

Cells were transfected with pALTER-TUBB3 or siRNA-tubb3. They were then exposed to STX140, STX243, paclitaxel, STX66, ENMD1198, vinorelbine or colchicine for 4 days and the IC_50_ values were obtained from a graph of percent proliferation *vs* inhibitor concentration and calculated using Prism (Graphpad Software). ^***^P<0.001, ^**^P<0.01 and ^*^P<0.05.
